# 肠道真菌组在肺癌免疫治疗中的作用：机制、挑战与转化前景

**DOI:** 10.3779/j.issn.1009-3419.2026.101.07

**Published:** 2026-03-20

**Authors:** Xinyi WANG, Yu SUN, Min LIU, Zixuan HE, Wenjiao CAO, Weiying LIU

**Affiliations:** ^1^730000 兰州，兰州大学第一临床医学院; ^1^The First Clinical Medical College, Lanzhou University, Lanzhou 730000, China; ^2^730000 兰州，兰州大学第一医院呼吸与危重症一病区; ^2^The First Department of Respiratory and Critical Care Medicine, The First Hospital of Lanzhou University, Lanzhou 730000, China

**Keywords:** 肠-肺轴, 肺肿瘤, 肠道真菌, 免疫检查点抑制剂, 生物标志物, Gut-lung axis, Lung neoplasms, Gut mycobiome, Immune checkpoint inhibitors, Biomarkers

## Abstract

肺癌是全球发病率与死亡率最高的恶性肿瘤之一，免疫检查点抑制剂（immune checkpoint inhibitors, ICIs）显著改善晚期患者预后，但超半数患者获益有限。肠道微生物可通过肠-肺轴调控肿瘤免疫与ICIs疗效，肠道真菌组占比微小却具备关键免疫调控作用，相关研究远滞后于细菌。本文系统梳理肠道真菌组与肺癌的临床关联，阐明真菌组及其代谢物通过免疫调控、代谢调节及跨界互作影响ICIs疗效的潜在机制，总结饮食干预、粪便微生物移植、靶向真菌等潜在策略，为建立真菌组预测模型、开发精准干预手段、提升ICIs疗效提供理论支撑。

## Abstract (en)

Lung cancer is one of the most common malignancies with the highest morbidity and mortality worldwide. Immune checkpoint inhibitors (ICIs) have significantly improved the prognosis of patients with advanced lung cancer, but more than half of patients derive limited benefit. The gut microbiota can regulate tumor immunity and ICIs efficacy via the gut-lung axis. Although accounting for a small proportion, the gut mycobiome exerts key immunomodulatory effects, yet relevant research lags far behind that of bacteria. This review systematically summarizes the clinical association between the gut mycobiome and lung cancer, clarifies the potential mechanisms by which the mycobiome and its metabolites influence ICIs efficacy through immune regulation, metabolic modulation, and cross-kingdom interactions, and summarizes potential strategies including dietary intervention, fecal microbiota transplantation, and fungal targeting. It provides theoretical support for establishing mycobiome-based predictive models, developing precise interventions, and improving the efficacy of ICIs.

肺癌是全球最常见的癌症类型，也是癌症死亡的主要原因^[1]^，2022年我国肺癌新发病例占新发恶性肿瘤总数的22.2%，死亡病例占肿瘤死亡总数的28.5%^[2]^，其高发病率和高致死率对人类健康造成极大威胁，因此对肺癌的早期诊断与治疗的研究迫在眉睫。免疫检查点抑制剂（immune checkpoint inhibitors, ICIs）的出现使得肺癌的治疗获得了重大突破，特别是程序性死亡受体1（programmed cell death protein 1, PD-1）、程序性死亡配体1（programmed death ligand 1, PD-L1）和细胞毒性T淋巴细胞相关抗原4（cytotoxic T lymphocyte antigen 4, CTLA-4）抑制剂已成为晚期非小细胞肺癌（non-small cell lung cancer, NSCLC）的标准治疗之一，但仅有部分患者从中得到预后获益。另外，ICIs的耐药性及免疫相关不良反应（immune-related adverse events, irAEs）的发生也表明ICIs疗法具有局限性^[3]^。随着宏基因组技术的发展，“肠-肺轴”这一概念得以提出，研究^[4]^证实，肠道微生物组对ICIs疗效具有调节作用，其中特定菌群可以影响ICIs的响应度，并且与irAEs的发生率有关。这一发现提示，肠道微生物有望成为预测肺癌ICIs疗效的潜在生物标志物。

真菌仅占肠道微生物的0.1%，但其细胞壁上的独特细胞成分，可通过与宿主表面的模式识别受体（pattern recognition receptors, PRRs）结合，广泛参与宿主免疫调控。此外，肠道真菌还可产生多糖、毒素等代谢产物，调节宿主免疫细胞活性，影响肠道微生态平衡，对健康和疾病有显著的调节作用^[5]^。目前大多研究强调肠道细菌对肺癌免疫及ICIs疗效的影响，受限于早期测序技术对真菌检测灵敏度的不足，肠道真菌组的研究长期滞后于细菌研究。泛癌研究^[6,7]^证实，肠道真菌生态失调与结直肠癌、黑色素瘤等多种癌症有关，部分真菌可直接影响ICIs疗效，该结论尚未在肺癌人群中单独验证。但已有证据^[8]^显示，真菌可通过激活免疫通路或调节微生态互作影响肺癌进展，具备成为早筛标志物与免疫治疗靶点的巨大潜力。基于肠道真菌在免疫调节中的特殊地位，本综述旨在系统梳理肠道真菌组与肺癌免疫及ICIs疗效相关的最新证据，深入探讨其潜在机制，进一步探索肠道真菌组作为未来肺癌生物标志物的可能性，为肺癌的综合治疗拓宽视野。

## 1 肠道真菌组与肺癌免疫的临床关联

### 1.1 肺癌患者肠道真菌组特征

肠道真菌生态失调已经在多种癌症中有所体现，主要表现为丰度和多样性的变化，且具有肿瘤亚型特异性。与健康人群相比，肺腺癌患者的肠道真菌多样性和丰度均有所提升。在门水平上，肺腺癌患者体内子囊菌门（Ascomycota）丰度下降，担子菌门（Basidiomycota）丰度增加；属水平上，酵母菌属（Saccharomyces）和曲霉菌属（Aspergillus）及食霉菌属（Apiotrichum）显著富集，而念珠菌属（Candida）减少^[7]^。相比之下，肺鳞癌患者则呈现出芽生菌属（Blastomyces）丰度的显著增加^[6]^。然而，现有研究多为横断面分析，样本量有限，且不同研究间真菌谱差异较大，可能与样本处理、测序方法异质性有关，因此，推动测序方法及流程的标准化对厘清肠道真菌与肺癌之间的关联十分必要。

### 1.2 真菌作为ICIs疗效潜在生物标志物

当前临床常用的疗效预测生物标志物主要为PD-L1及肿瘤突变负荷（tumor mutational burden, TMB），均存在明显局限性。肿瘤组织PD-L1的表达具有时空异质性，具体表现为PD-L1的表达在同一肿瘤的不同位置可能不同，随着病程进展，肿瘤PD-L1表达可能也会发生动态变化。而TMB的评估检测成本高，阈值鉴定存在争议，无法解释所有临床现象，因此，探索肠道真菌组这类新型补充标志物尤为必要。越来越多的研究证实，肠道真菌组在ICIs治疗中发挥重要作用，可能作为肺癌ICIs治疗反应的调节剂和预测因子，根据其效应类型分为有益与有害真菌两大类（表1）^[6,7,9,10]^。

**表1:** 肠道真菌组对NSCLC的效应及临床关联

Effect type	Fungal groups	Clinical relevance	References
Beneficial effect	Aspergillus pseudonomiae, Fusarium flagelliforme, Schizosaccharomyces japonicus	Improve the efficacy of ICIs therapy, increase the proportion of patients with 6-mon PFS, prolong PFS and OS	^[9]^
	Cutaneotrichosporon cutaneum, Rozelomycota	Reduce the irAEs in NSCLC patients, maintain immune homeostasis	^[10]^
	Hymenoscyphus immutabilis, Clavulinopsis fusiformis	Associated with high PD-L1 expression, synergistically enhance the anti-tumor immunity	^[10]^
Adverse effect	Cortinarius davemallochii, Helotiales, Chaetospheriales, Tremelomycetes	Overexpression associated with shortened OS (≤6 mon) in NSCLC patients	^[10]^
	Saccharomyces cerevisiae	High abundance associated with impaired ICIs efficacy	^[10]^
	Thelephoraceae	Positivity associated with increased irAEs incidence in NSCLC patients	^[10]^
	Apiotrichum	Significantly enriched in the gut of patients with lung adenocarcinoma, increased abundance linked to tumor progression	^[7]^
	Blastomyces	Increased intestinal abundance in lung squamous cell carcinoma patients correlated with tumor progression	^[6]^

NSCLC: non-small cell lung cancer; ICIs: immune checkpoint inhibitors; PFS: progression-free survival; OS: overall survival; irAEs: immune-related adverse events; PD-L1: programmed death ligand 1.

#### 1.2.1 有益真菌

有益真菌主要通过调控抗肿瘤免疫反应、优化肿瘤微环境（tumor microenvironment, TME），或与肠道细菌协同作用，提升ICIs治疗响应并延长患者生存期。一项多队列荟萃分析研究^[9]^识别出23种真菌与NSCLC ICIs的有效性相关，而17种真菌与6个月无进展生存期（progression-free survival, PFS）相关，其中9种包含于前文所述23种真菌之中，研究表明，Aspergillus pseudonomiae的高丰度与ICIs有效性提高相关，可提高患者6个月PFS比率，并延长PFS及总生存期（overall survival, OS），是目前明确的ICIs疗效正向调节真菌。同时，日本裂殖酵母（Schizosaccharomyces japonicus）及鞭形镰刀菌（Fusarium flagelliforme）丰度增加也表现出类似的积极趋势。此外，Dora等^[10]^的研究证实，罗兹菌门（Rozellomycota）和皮状丝孢酵母（Cutaneotrichosporon cutaneum）类群在未发生irAEs的NSCLC患者中富集，提示其可能通过维持免疫稳态，降低治疗毒性，间接保障ICIs治疗的有效性。此研究还观察到，在PD-L1表达水平较高（≥50%）的肿瘤患者体内，难变膜盘菌（Hymenoscyphus immutabilis）和梭形黄拟锁瑚菌（Clavulinopsis fusiformis）更为丰富，这表明真菌可能与肿瘤免疫微环境之间存在关联，与免疫检查点分子共同调控肿瘤免疫。

一项研究^[11]^确定了与ICIs治疗反应相关的基于肠道真菌组的两种不同肠道类型，即有利肠型和不利肠型，以与免疫治疗结果相关的不同微生物组成为特征。有利肠型的特征为：较高的真菌与细菌α多样性、较高的担子菌门/子囊菌门（Ascomycota）比值、产丁酸菌（Butyrate-producing bacteria）的增加及丁酸与糖和淀粉代谢相关通路的显著富集。进一步验证发现，有利肠型患者TME中CD8^+^ T细胞浸润率更高，且表现出更高的ICIs反应率和总体生存率。在预测效能方面，Huang等^[12]^的研究显示，在接受免疫治疗的包括NSCLC在内的多瘤种患者中，真菌作为生物标志物[曲线下面积（area under the curve, AUC）=0.87]相比细菌（AUC=0.83）而言，具有更高的疗效预测性，二者联合模型进一步提高了预测准确性（AUC=0.89）。但真菌与ICIs疗效的关联多基于多瘤种分析，肺癌特异性验证不足，未来需要开展肺癌特异性队列研究进一步验证；联合预测模型的临床转化还需解决样本处理标准化、检测成本控制等挑战。

#### 1.2.2 有害真菌

有害真菌可能通过诱导免疫抑制、破坏肠道微生态平衡，降低ICIs疗效并缩短患者生存期，部分类群还与治疗毒性密切关联。研究^[10]^证实，NSCLC患者肠道中真菌类群Cortinarius davemallochii、刺球壳目（Chaetosphaeriales）、银耳纲（Tremellomycetes）以及柔膜菌目（Helotiales）的过度表达与较短的OS（≤6个月）显著关联，革菌科（Thelephoraceae）阳性者与irAEs发生相关。而有趣的是，在晚期NSCLC患者中，肠道真菌组α多样性高于细菌群落与OS缩短有关，这表明有害真菌过度定植可能是ICIs疗效的负面调节因素，该关联性在PD-L1阴性患者中尤为显著，提示其作为特定人群疗效预测指标的潜力。此外，另一项研究^[9]^发现酿酒酵母（Saccharomyces cerevisiae）的高丰度与ICIs疗效受损有关。

综上所述，现有研究充分凸显出肠道真菌组具备作为预测ICIs疗效及评估治疗相关毒性生物标志物的潜力，未来研究应着重解决现有成果在肺癌特异性验证和临床转化方面面临的挑战，以推动这一领域向临床应用方向发展。

## 2 肠道真菌对肺癌免疫的影响机制

肠道真菌组主要通过免疫调节机制、代谢产物机制及跨界调控机制，影响肿瘤免疫微环境及ICIs疗效，进而影响肺癌进展。

### 2.1 免疫调控机制

肠道真菌主要通过激活先天免疫途径调节宿主免疫系统功能，其核心机制是真菌病原体相关分子模式（pathogen-associated molecular pattern molecules, PAMPs）与宿主细胞表面的PRRs结合，触发下游信号级联反应，进而调控免疫细胞的活化及功能。真菌PAMPs主要包括β-葡聚糖、α-甘露聚糖、几丁质等^[13]^，而PRRs则主要包括Dectin-1、Dectin-2、Mincle等C型凝集素受体^[14]^，这些受体在免疫细胞[如巨噬细胞、树突状细胞、自然杀伤（natural killer, NK）细胞]表面广泛表达，是介导真菌免疫应答的关键分子（表2）^[8,15-19]^。

**表2:** 肠道真菌组对肺癌的潜在免疫调控机制

PRRs	PAMPs	Signaling pathways	Effects	References
Dectin-1	A. sydowii-derived β-glucan	β-glucan/Dectin-1/CARD9	Immunosuppression: Activates MDSCs, reduces T-cell cytotoxicity, and promotes the accumulation of PD-1^+^CD8^+^ T cells	^[8]^
	Yeast-derived β-glucan	Dectin-1/SYK/CARD9/Erk	Immunostimulation: Converts immunosuppressive TAMs to a pro-inflammatory M1 phenotype	^[15]^
	Yeast-derived β-glucan	Dectin-1/MS4A4A	Activates NK cells, enhances anti-tumor and metastasis control	^[16]^
	Yeast-derived β-glucan	Mediates B cell differentiation-related signals	Immunostimulation: Promotes B-cell differentiation, costimulatory molecule expression and Ig production; combined with anti-PD-1, increases CD19^+^ B-cell infiltration in TME	^[17]^
Dectin-2	α-mannan	Mainly via signaling mediated by agonist BDC-3042	Reprograms TAMs to pro-inflammatory phenotype; enhances antigen presentation and T cell-mediated anti-tumor immunity	^[18]^
Mincle	Fungal cell wall glycolipids	Mincle/Syk/NF-κB	Maintains TAMs protumor activity and drives cancer progression	^[20]^

PRRs: pattern recognition receptors; PAMPs: pathogen-associated molecular pattern molecules; MDSCs: myeloid-derived suppressor cells; TAMs: tumor-associated macrophages; TME: tumor microenvironment; NF-κB: nuclear factor kappa B; PD-1: programmed death protein 1.

Dectin-1是一种C型凝集素受体，是癌症免疫中关键的真菌感应受体，在癌症免疫中发挥重要作用，且对肿瘤免疫的影响具有高度的背景依赖性，同一受体可以诱导免疫抑制作用或免疫刺激级联反应，最终结果由配体类型和细胞环境决定，该双重作用特征目前主要基于动物模型验证。在肺癌小鼠模型中，Dectin-1介导的通过β-葡聚糖/Dectin-1/CARD9信号对聚多曲霉（Aspergillus sydowii）的识别诱导髓源性抑制细胞（myeloid-derived suppressor cells, MDSCs）活化，降低T细胞的细胞毒性，并促进PD-1^+^CD8^+^ T细胞的积累，进一步导致免疫抑制。在患者样本中发现，聚多曲霉的高丰度与肿瘤浸润的MDSCs、调节性T细胞（regulatory T cells, Tregs）和PD-1^+^CD8^+^ T细胞的增加呈正相关^[8]^。相反，Dectin-1与酵母衍生的β-葡聚糖结合，可以通过Dectin-1/SYK/CARD9/Erk途径，将免疫抑制性肿瘤相关巨噬细胞（tumor-associated macrophages, TAMs）转化为具有强大免疫刺激活性的类M1表型，从而增强抗肿瘤免疫^[15]^。Mattiola等^[16]^的研究表明，Dectin-1与巨噬细胞脂筏内的四跨分子MS4A4A相互作用，可以激活NK细胞，在抗肿瘤及控制肿瘤转移方面至关重要。MS4A4A缺失细胞模型中，Dectin-1信号传导受损，导致促炎介质的产生减少，NK细胞介导的抗肿瘤活性降低。此外，Bai等^[17]^研究表明，β-葡聚糖诱导Dectin-1活化促进血浆B细胞的分化、共刺激分子的表达和免疫球蛋白的产生，增强抗肿瘤免疫反应。在Lewis肺癌小鼠模型中，β-葡聚糖联合抗PD-1治疗增加了TME中CD19^+^ B细胞的浸润（提升1.75倍），扩大了生发中心B细胞，并提高了抗肿瘤效果，延缓肿瘤进展^[17]^。这一发现将Dectin-1的已知免疫刺激作用扩展到T细胞介导的免疫之外，突出了其在协调涉及多种免疫细胞类型的全面抗肿瘤反应方面的潜力。

除Dectin-1外，Dectin-2作为另一种重要的真菌感应受体，在肠道真菌组调控肺癌ICIs治疗中的作用已获得初步临床数据支撑。临床前动物模型显示，Dectin-2激动剂BDC-3042可以将TAMs从免疫抑制表型重新编程为促炎表型，增强抗原呈递并促进T细胞介导的抗肿瘤反应。早期临床试验^[18]^结果证实，该疗法在NSCLC患者中具有良好的耐受性，并表现出免疫刺激作用，体现出其与ICIs的联合应用潜力。

Mincle是另一种重要的C型凝集素受体，其配体主要为真菌细胞壁中的糖脂类物质^[20]^。相关机制研究主要基于人类肺癌队列证据，通过单细胞RNA测序发现，模式识别受体Mincle主要在NSCLC患者的M2样TAMs中高度表达，通过Mincle/SYK/核因子-κB（nuclear factor-κB, NF-κB）信号通路，促进TAMs驱动的癌症进展。研究^[19]^显示，Mincle^+ ^TAM亚组与NSCLC患者的死亡率显著相关，Mincle表达升高与预后不良、T细胞浸润减少和肿瘤进展增强相关。

### 2.2 代谢产物机制

肠道真菌可通过分解各类底物产生多种代谢产物，包括多糖、毒素、多元醇、酶类等，其中研究最多的为β-葡聚糖，直接或间接调控肺癌的发生发展及ICIs治疗效应。真菌代谢产物的作用具有双重性，既可能通过诱导DNA损伤、破坏肠道屏障功能、抑制宿主免疫反应、增强ICIs耐药性及与细菌发生促肿瘤相互作用等方式促进肿瘤进展；也可通过诱导肿瘤细胞凋亡、抑制肿瘤细胞增殖、调节宿主免疫系统功能及增强抗肿瘤免疫反应等途径发挥抗癌作用^[21]^。

#### 2.2.1 多糖类代谢产物

β-葡聚糖作为真菌来源的代表性多糖类代谢产物，已被证实具有强大的免疫调节活性，其通过与Dectin-1等PRRs结合，激活下游免疫信号通路，调控巨噬细胞、T细胞、B细胞及NK细胞等多种免疫细胞的功能，增强抗肿瘤免疫反应。研究^[22]^证实，β-葡聚糖的结构因其来源不同而异，进而导致不同的免疫反应。例如，来自白色念珠菌（Candida albicans）的β-葡聚糖激活Dectin-1，已被证明可以通过促进PD-L1的核转位和随后CXCL1和CXCL2的分泌来上调中性粒细胞中的PD-L1，从而导致局部免疫抑制表型^[23]^。而酵母来源的β-葡聚糖可以通过NF-κB/Nrf2通路降低Nrf2蛋白水平，改善癌症药物耐药性^[24]^。此外，从桦褐孔菌（Inonotus obliquus）中分离的多糖被证实可以通过抑制丝裂原活化蛋白激酶（mitogen-activated protein kinases, MAPKs）、磷脂酰肌醇-3-激酶/蛋白激酶B（phosphatidylinositol-3-kinase/protein kinase B, PI3K/Akt）和NF-κB信号通路，降低基质金属蛋白酶（matrix metalloproteinase, MMP）-2和MMP-9的表达水平和活性，从而抑制人类肺癌的侵袭和迁移^[25]^。

#### 2.2.2 毒素类代谢产物

部分肠道真菌可产生毒素类代谢产物，如黄曲霉素^[26]^、赭曲霉毒素^[27]^，这些毒素具有较强的致癌性，可能通过损伤DNA、诱导慢性炎症和免疫失调、促进基因突变等方式促进肺癌的发生发展。桔青霉素（Citrinin, CIT）作为一种从聚酮类中提取的霉菌毒素，可由各种真菌菌株产生[包括红曲霉菌（Monascus purpureus）、曲霉属（Aspergillus）等]，虽然人类队列研究尚无相关临床报道，但CIT诱导细胞死亡、降低DNA修复能力以及诱导癌细胞毒性和基因毒性作用的潜力已在细胞实验中得到初步验证^[28]^。目前真菌毒素与肺癌的关联研究仍处于初步探索阶段，未来需开展肺癌特异性队列研究及干预性实验，进一步明确其作用及机制。

#### 2.2.3 其他衍生代谢物

除常见代谢产物外，还有部分衍生代谢物显现出显著的抗癌特性。例如，来自血红丛赤壳（Nectria haematococca）的真菌免疫调节蛋白，通过抑制PI3K/Akt途径抑制体内肺癌细胞生长^[29]^。此外，解脂耶氏酵母（Yarrowia lipolytica）产生的新型L-天冬酰胺酶显示可诱导A549细胞的自噬激活，抑制肺癌细胞的增殖和迁移^[30]^。

### 2.3 跨界调控机制

真菌-细菌互作已经被证明在多个领域具有核心重要性^[31]^。在肺癌免疫中，真菌从来不是孤立运作的，真菌群落可以通过与细菌、病毒的协同或拮抗作用，影响宿主免疫。Narunsky-Haziza等^[32]^观察到多种癌症类型中真菌和细菌的多样性、丰度及共现率之间存在强烈正相关。Seelbinder等^[33]^的研究结果表明，在肺癌患者中，产乳酸盐细菌（Lactic acid bacteria）的增加与产短链脂肪酸细菌的减少可以驱动肠道念珠菌（Candidiasis）的扩张，乳酸积累使念珠菌过度定植，从而产生各种代谢物，破坏上皮屏障，导致癌症的发生。由此可知，补充短链脂肪酸可能改变肠道生态位，起到一定的抗肿瘤作用。在结肠癌患者中，丁酸盐可通过NF-κB/GNLY轴激活CD8^+^ T细胞，显著增强抗PD-1治疗的疗效^[34]^。此研究提示在肺癌中可能通过类似机制增强ICIs疗效，为ICIs联合短链脂肪酸补充治疗策略提供了理论依据。对于真菌与病毒的互作，目前的研究十分有限，它们可能通过基因转移、协同调节宿主免疫或生态位竞争，共同塑造TME并影响免疫治疗疗效。

真菌与细菌通过跨界互作共同塑造TME，孤立干预单一菌群难以实现免疫稳态重建，因此，在后续临床试验及应用中必须以真菌-细菌互作为核心，构建真菌-细菌-病毒生态网络，而非孤立靶向真菌。

## 3 基于肠道真菌组的肺癌临床干预策略

### 3.1 经消化道饮食调节

膳食纤维可被肠道菌群发酵而产生短链脂肪酸，而短链脂肪酸可能通过真菌-细菌互作机制改变肠道生态位，从而辅助肺癌的治疗。Buttar等^[35]^研究发现，28 d真菌饮食（富含纤维、抗性淀粉、全食物，避免加工高糖食物）可使肠道念珠菌丰度下降72.4%，同时增加有益真菌比例。一项涵盖3236人的中国前瞻性队列研究^[36]^进一步证实，饮食多样性与肠道菌群α多样性呈正相关，尤其是谷物、蔬菜、豆制品的均衡摄入，且可通过调节粪便代谢物间接影响真菌-细菌平衡，为中国肺癌患者饮食干预提供了人群特异性依据。需注意的是，中国部分地区高油高盐烹饪方式可能削弱膳食纤维的正向作用，干预方案需针对性调整。

补充益生菌已经被证明可以对癌症免疫产生正向影响^[37]^，一项针对118例接受ICIs治疗的晚期NSCLC患者的回顾性研究^[38]^显示，ICIs治疗后接受丁酸梭菌（Clostridium butyricum）补充剂治疗，PFS和OS显著延长。而益生菌能够影响肠道真菌种类的丰富度，研究^[39]^表明，每天服用益生菌的受试者肠道真菌的丰度和Shannon多样性明显低于仅按处方服用益生菌的受试者；因此，探索益生菌影响肠道真菌的潜在机制、补充益生真菌和下游代谢物，制定真菌-细菌协同调节的饮食干预方案以及通过特定阻断营养代谢来对抗有害真菌，在抗癌治疗方面同样具有巨大潜力。目前缺乏直接针对“真菌靶向”益生菌制剂对肺癌ICIs疗效影响的临床试验证据，因此，在未来的研究中，探索真菌及其营养代谢的机制，对肺癌的辅助治疗意义深远。

酵母衍生的β-葡聚糖可以通过多种信号级联调节先天性免疫和适应性免疫，增强抗肿瘤免疫力^[21]^。在各种临床前模型中，口服β-葡聚糖已被证明可以通过增加巨噬细胞和NK细胞的比例和增加其功能活性来延缓肿瘤进展，NK细胞耗竭降低β-葡聚糖在携带肿瘤小鼠中的治疗效果^[40]^。Lewis肺癌小鼠模型中，β-葡聚糖逆转了由肿瘤衍生因子诱导的树突状细胞功能障碍，促进了Th1细胞分化和细胞毒性T淋巴细胞激活，并改善了抗肿瘤反应。此外，β-葡聚糖诱导多形核MDSCs凋亡，促进单核MDSCs分化为能够激活CD4^+^和CD8^+ ^T细胞的成熟抗原提呈细胞^[41]^。值得注意的是，不同的给药方式还可以影响β-葡聚糖激活补体的途径，口服时，通过C3/iC3b轴激活旁路途径，从而激活中性粒细胞，杀伤被iC3b标记的肿瘤细胞，而静脉注射时主要通过形成循环免疫复合物，激活经典补体途径，增强抗体依赖性细胞吞噬作用，提升活性氧水平，继而发挥抗肿瘤作用^[21,42]^。上述证据表明，β-葡聚糖可能是抗肿瘤的强大免疫调节因子，以及ICIs治疗中不可或缺的一部分。

此外，使用氟康唑等唑类药物选择性清除有害真菌，可能重塑免疫抑制性TME^[43]^。在安全性评估方面，需特别警惕非选择性抗真菌治疗的风险，非选择性抗真菌治疗可能破坏肠道微生态平衡，精准靶向有害真菌的制剂尚未开发，其与ICIs联合使用的时机、剂量及安全性仍需探索。这警示我们在通过抗真菌或粪便微生物移植等手段调节菌群时，需密切关注其对免疫稳态的潜在影响，以避免诱发或加重irAEs。

### 3.2 粪便微生物移植

粪便微生物移植是将健康人群粪便中的功能菌群移植入患者肠道内，通过重建患者肠道微生态系统，从而治疗某些特定疾病。患者的肠道微生物菌群会影响ICIs的疗效，对ICIs的初级耐药性可归因于肠道微生物组组成异常。Routy等^[44]^的研究表明，对ICIs无应答小鼠进行粪便微生物移植后口服补充嗜黏蛋白阿克曼菌（A. muciniphila）可通过增加小鼠肿瘤组织中T淋巴细胞的募集，以白细胞介素（interleukin, IL）-12依赖的方式恢复抗PD-1的疗效。在真菌中仍有类似的证据，Hu等^[11]^的研究发现，来自有利肠型供体的粪便微生物移植增强了对抗PD-1/PD-L1治疗的反应。因此，在未来的研究中，有必要针对肺癌患者进行粪便微生物移植的大样本临床试验，供体筛选需结合中国人群饮食相关的菌群特征，优先选择有利肠型供体，避免直接套用西方供体标准。

## 4 挑战与展望

肠道真菌组是影响肺癌ICIs疗效的一个极具潜力但研究尚浅的新兴靶点，该领域的转化应用仍受限于技术与机制层面的多重壁垒。首先是测序与分析的标准化难题，由于肠道真菌生物量极低，DNA提取过程困难且极易受环境污染干扰，内部转录间隔区（internal transcribed spacer, ITS）扩增子测序在引物选择及生物信息分析上缺乏统一标准，导致不同研究间的结果难以横向比较^[45]^。同时，现有真菌数据库覆盖度不足，宏基因组分析中真菌序列常被海量细菌数据掩盖，极大增加了物种精准注释的难度。其次是因果机制解析的复杂性，目前研究多停留在相关性层面，临床干预研究样本量小，肺癌特异性数据不足，缺乏对真菌及其特定代谢产物调节ICIs疗效的确切分子机制验证。此外，真菌-细菌的跨界互作网络极其复杂，如何明确真菌对肺癌免疫微环境的独立影响与协同效应，仍是亟待解决的关键问题。

未来，应开展以生物标志物为导向的大样本、前瞻性临床试验以及多中心随机对照试验验证真菌组标志物的诊断与疗效预测价值，优化细菌-真菌联合预测模型。其次，应深入关于机制的研究，探索干预核心靶点。最后，开发精准干预策略，包括靶向真菌-免疫通路的小分子药物、个性化粪便微生物移植、真菌饮食与益生真菌制剂，同时通过随机对照试验评估其与ICIs联合使用的有效性和安全性，最终实现肺癌ICIs治疗效益的最大化。
